# Evaluation of in silico predictors on short nucleotide variants in *HBA1*, *HBA2*, and *HBB* associated with haemoglobinopathies

**DOI:** 10.7554/eLife.79713

**Published:** 2022-12-01

**Authors:** Stella Tamana, Maria Xenophontos, Anna Minaidou, Coralea Stephanou, Cornelis L Harteveld, Celeste Bento, Joanne Traeger-Synodinos, Irene Fylaktou, Norafiza Mohd Yasin, Faidatul Syazlin Abdul Hamid, Ezalia Esa, Hashim Halim-Fikri, Bin Alwi Zilfalil, Andrea C Kakouri, ClinGen Hemoglobinopathy Variant Curation Expert Panel, Marina Kleanthous, Petros Kountouris

**Affiliations:** 1 Molecular Genetics Thalassaemia Department, The Cyprus Institute of Neurology and Genetics Nicosia Cyprus; 2 Leiden University Medical Center Leiden Netherlands; 3 Centro Hospitalar e Universitário de Coimbra Coimbra Portugal; 4 Laboratory of Medical Genetics, National and Kapodistrian University of Athens Athens Greece; 5 Division of Endocrinology, Metabolism and Diabetes, First Department of Pediatrics, National and Kapodistrian University of Athens Athens Greece; 6 Haematology Unit, Cancer Research Centre, Institute for Medical Research, National Health of Institutes (NIH), Ministry of Health Malaysia Selangor Malaysia; 7 Malaysian Node of the Human Variome Project, School of Medical Sciences, Health Campus, Universiti Sains Malaysia Kelantan Malaysia; 8 Human Genome Centre, School of Medical Sciences, Health Campus, Universiti Sains Malaysia Kelantan Malaysia; The Ohio State University United States; Icahn School of Medicine at Mount Sinai United States

**Keywords:** variant classification, in silico prediction, haemoglobinopathies, globin genes, thalassaemia, Human

## Abstract

Haemoglobinopathies are the commonest monogenic diseases worldwide and are caused by variants in the globin gene clusters. With over 2400 variants detected to date, their interpretation using the American College of Medical Genetics and Genomics (ACMG)/Association for Molecular Pathology (AMP) guidelines is challenging and computational evidence can provide valuable input about their functional annotation. While many in silico predictors have already been developed, their performance varies for different genes and diseases. In this study, we evaluate 31 in silico predictors using a dataset of 1627 variants in *HBA1*, *HBA2,* and *HBB*. By varying the decision threshold for each tool, we analyse their performance (a) as binary classifiers of pathogenicity and (b) by using different non-overlapping pathogenic and benign thresholds for their optimal use in the ACMG/AMP framework. Our results show that CADD, Eigen-PC, and REVEL are the overall top performers, with the former reaching moderate strength level for pathogenic prediction. Eigen-PC and REVEL achieve the highest accuracies for missense variants, while CADD is also a reliable predictor of non-missense variants. Moreover, SpliceAI is the top performing splicing predictor, reaching strong level of evidence, while GERP++ and phyloP are the most accurate conservation tools. This study provides evidence about the optimal use of computational tools in globin gene clusters under the ACMG/AMP framework.

## Introduction

With genetic testing frequently employed by clinical laboratories to aid diagnosis and treatment decisions in different diseases (Richards et al., 2015), advances in sequencing technology produce an excessive amount of sequencing data leading to a rapidly enlarging pool of new unclassified variants. While sequencing data provide new candidates for therapeutic interventions and personalised medicine, they also introduce challenges in correctly classifying variants as pathogenic or benign. Thus, variant interpretation often relies on human expertise to gather information from different and diverse sources as to combine individual pieces of evidence into a comprehensive estimate with high confidence (Luo et al., 2019).

To assist in the establishment of a common framework for standardised variant classification, the American College of Medical Genetics and Genomics (ACMG) and the Association for Molecular Pathology (AMP) published joint recommendations for the interpretation of genetic variants (Richards et al., 2015). The ACMG/AMP framework was designed for use across different genes and diseases, thus requiring further specification in disease-specific scenarios. In response to this need, the Clinical Genome (ClinGen) Resource formed various disease-specific variant curation expert panels (VCEPs) to develop specifications to the ACMG/AMP framework (Rehm et al., 2015). The ClinGen Haemoglobinopathy VCEP focuses on performing and testing the applicability of haemoglobinopathy-specific modifications to the standard ACMG/AMP framework before proceeding with the classification and interpretation of variants related to haemoglobinopathies (Kountouris et al., 2021). Haemoglobinopathies represent the commonest groups of inherited monogenic disorders affecting approximately 7% of the global population (Cao and Kan, 2013). They are caused by genetic defects in genes located in the α-globin locus (Accession: NG_000006) and in the β-globin locus (Accession: NG_000007). To date, there are over 2400 different naturally occurring globin gene variants, which are collected and manually curated in IthaGenes, a haemoglobinopathy-specific database on the ITHANET portal (Kountouris et al., 2014).

The ACMG/AMP guidelines propose the use of in silico predictors (namely criteria PP3 and BP4 for pathogenic and benign evidence, respectively) as supporting evidence for variant pathogenicity classification (Richards et al., 2015). Several tools have already been developed to predict the impact of genetic variants and their relation to developing diseases. These tools fall into four main categories based on the theoretical background and type of data they use for predicting variant effect, namely sequence conservation-based, structure-based analysis, combined (i.e., including both sequence and structural features), and meta-predictors (Li and Wang, 2017).

The performance of different in silico tools varies across genes and diseases as numerous studies illustrated discrepancies regarding variant pathogenicity prediction (Ernst et al., 2018; Fortuno et al., 2018; Luo et al., 2019; Masica and Karchin, 2016; Pshennikova et al., 2019). Previous studies have also evaluated the performance of in silico predictors for globin gene variants (AbdulAzeez and Borgio, 2016; Tchernitchko et al., 2004), demonstrating a high degree of discordance between in silico tools. Therefore, it is evident that a disease- or gene-specific evaluation of in silico tools can provide evidence for the optimal selection or combination of tools to identify the functional impact of variants. Recently, ClinGen published a study on the performance of four in silico predictors using a set of 237 variants (Wilcox et al., 2021), suggesting that custom thresholds should be explored for each in silico tool to establish PP3 and BP4 criteria. However, given the impact of in silico tools on variant classification, further calibration with larger datasets is still needed to optimise their performance.

The main purpose of this study is to compare the performance of various in silico predictors and determine the most appropriate ones for predicting the functional impact of short nucleotide variants (SNVs) in *HBA1*, *HBA2*, and *HBB* related to haemoglobinopathies. To our knowledge, this is the largest comparative study of in silico tools for SNVs related to haemoglobinopathies in terms of both the number of tools used and the size of utilised variant dataset.

## Results

**Figure 1.**
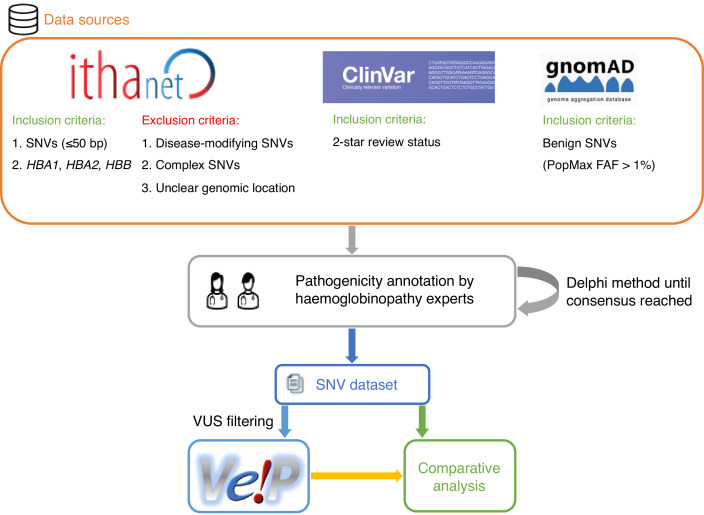
A schematic flowchart of the methodology followed for this comparative analysis.

We selected 31 in silico predictors, including those recommended by ClinGen (Rehm et al., 2015) and linked in the Variant Curation Interface (VCI) (Preston et al., 2022), along with additional tools described in literature. A total of 1627 SNVs were retrieved from the IthaGenes database (Kountouris et al., 2017; Kountouris et al., 2014) and were annotated using a Delphi approach with respect to their pathogenicity by experts (co-authoring this study) involved in haemoglobinopathy molecular diagnosis in five different countries. The annotated pathogenicity of each SNV was then used to evaluate its predicted pathogenicity provided by in silico tools. A detailed description of the overall methodology is provided in Materials and methods and illustrated in Figure 1.

**Figure 1—figure supplement 1.**
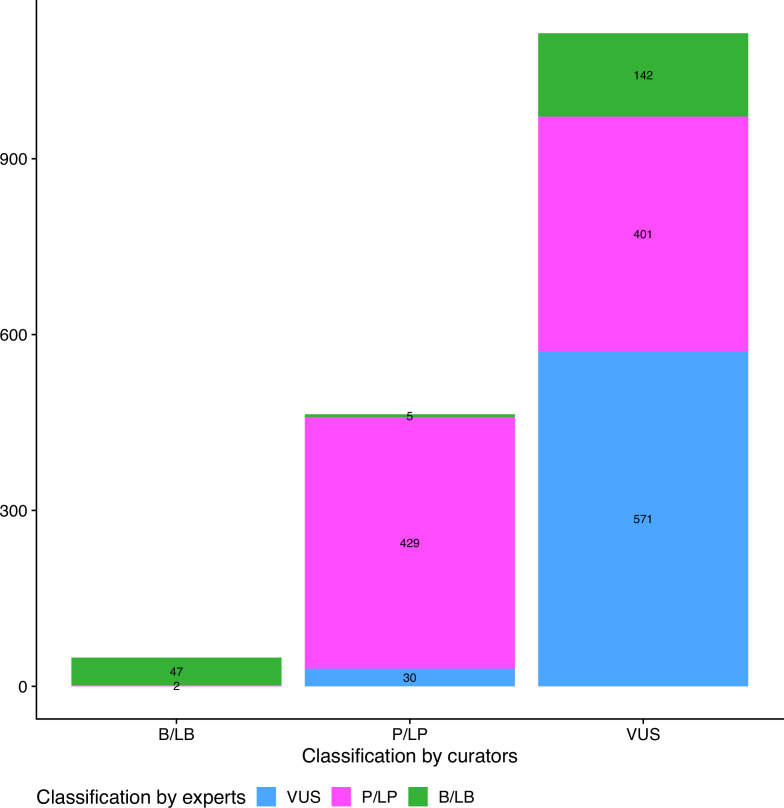
Comparison of initial and final pathogenicity classification of variants in the dataset.

### Descriptive analysis

**Figure 2.**
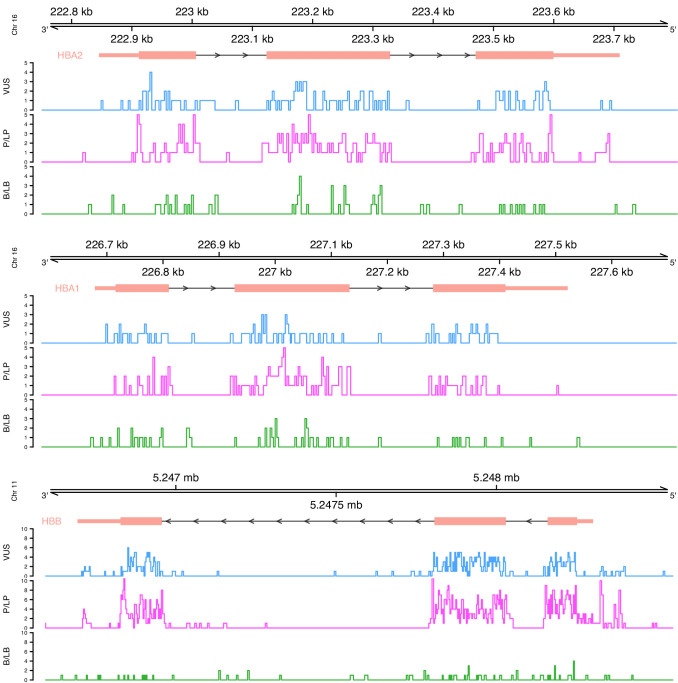
Distribution of variants on each globin gene based on their actual pathogenicity. A bin size of 3 bp (inframe) and 5 bp in exonic and intronic regions, respectively, is used for the illustration.

Initially, we performed a descriptive analysis of the full dataset, including variants annotated as variants of uncertain significance (VUS), which comprised 1627 SNVs. In terms of the annotated pathogenicity, 194 (11.9%) SNVs classified as benign/likely benign (B/LB), 832 (51.1%) as pathogenic/likely pathogenic (P/LP), and 601 (36.9%) as VUS. The distribution per globin gene is the following: 553 P/LP, 77 B/LB, and 403 VUS for *HBB* (total: 1033 SNVs; 63.5%), 173 P/LP, 66 B/LB, and 111 VUS for *HBA2* (total: 350 SNVs; 21.5%), and 106 P/LP, 51 B/LB, and 87 VUS for *HBA1* (total: 245 SNVs; 15%). Figure 2 illustrates the distribution of variants on each globin gene based on their annotated pathogenicity and demonstrates the highest fraction of P/LP variant in protein coding regions and in canonical splice sites. Increased numbers of P/LP variants are also observed in specific noncoding regions of the globin genes, such as polyadenylation regions and the promoter and 5’ UTR for *HBB*.

**Figure 3.**
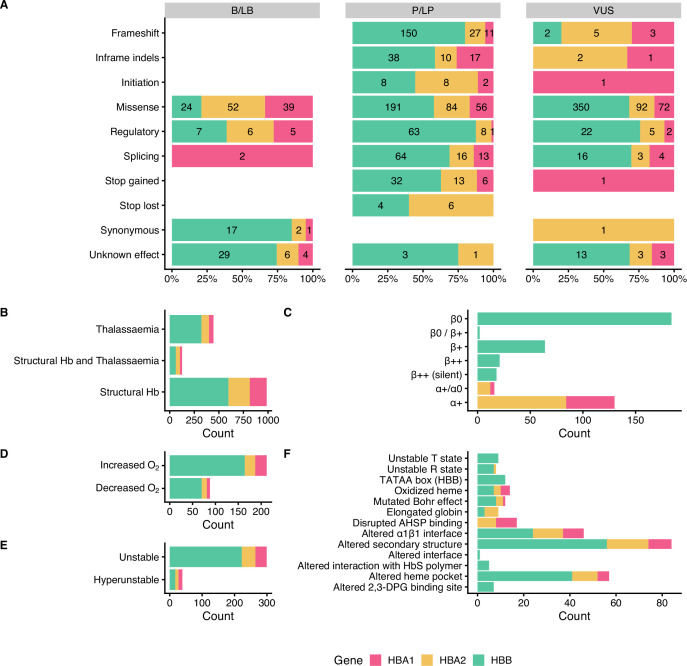
Descriptive plots of the short nucleotide variant (SNV) dataset. (**A**) Variant effect on gene/protein function with respect to the annotated pathogenicity status. (**B**) Haemoglobinopathy group, (**C**) thalassaemia phenotype, (**D**) O_2_ affinity, (**E**) Hb stability, and (**F**) molecular mechanisms.

Figure 3 summarises the distribution of SNVs in the dataset according to their effect on gene/protein function with respect to the annotated pathogenicity (Panel A), the annotated haemoglobinopathy group (Panel B), the thalassaemia allele phenotype (Panel C), altered oxygen affinity (Panel D), altered stability (Panel E), and the molecular mechanism involved in pathogenesis (Panel F). The effect on gene/protein function includes the following categories: (a) missense variants (SO:0001583), (b) synonymous variants (SO:0001819), (c) frameshift (SO:0001589), (d) initiation codon (SO:0000318), (e) in-frame indels (SO:0001820), (f) splicing, including cryptic splice site (SO:0001569), splice acceptor (SO:0001574), splice donor (SO:0001575) and splice region variants (SO:0001630), (g) stop lost (SO:0001578), (h) stop gained (SO:0001587), and (i) variants in regulatory elements, including promoter (SO:0001631), 5’ UTR (SO:0001623), 3’ UTR (SO:0001624), and polyadenylation variants (SO:0001545). Importantly, there are no B/LB null variants (i.e., frameshifts, stop gained, canonical splice sites, initiation codon) in the dataset, which reflects that loss-of-function is a primary disease mechanism, particularly for thalassaemia syndromes. In contrast, missense variants, representing the largest variant type category (total: 960 SNVs; 59%), are present in all pathogenicity categories, with 115 (12% of SNVs in the category), 331 (34.5%), and 514 (53.5%) annotated as B/LB, P/LP, and VUS, respectively. The distribution of missense variants in the three categories and the high percentage of missense VUS highlight the challenge to interpret the pathogenicity of missense variants in the globin genes, requiring rigorous study of available evidence, including computational evidence.

Moreover, the dataset comprises SNVs causing structural haemoglobinopathies (986 SNVs), thalassaemia (445 SNVs), and both thalassaemia and structural haemoglobinopathies (128 SNVs). The thalassaemia phenotype group describes the allele phenotype and includes *HBA1* and *HBA2* variants (α^+^/α^0^ and α^+^; total: 146 SNVs) and *HBB* variants (β^0^, β^0^/β^+^, β^+^, β^++^ (silent) and β^++^; total: 289 SNVs). Here, we observed that most variants have allele phenotype of α^+^ (130 SNVs) and β^0^ (184 SNVs). The category of Hb stability is further divided into hyperunstable (39 SNVs) and unstable (299 SNVs), while the Hb O_2_ affinity group is divided into increased O_2_ affinity (212 SNVs) and decreased O_2_ affinity (88 SNVs). The main molecular mechanisms disrupted are alterations of the secondary structure (84 SNVs), heme pocket (57 SNVs), and α1β1 interface (46 SNVs). The disruption of the molecular mechanisms has been associated with clinical phenotypes, such as haemolytic anaemia, reticulocytosis, erythrocytosis, and cyanosis (Thom et al., 2013).

### Evaluation of in silico tools as binary predictors

Table 1 shows a comparison of all in silico predictors used in this study as binary classifiers of pathogenicity, against the consensus dataset with VUS removed. For each tool, we varied the decision threshold for the whole range of possible prediction scores and calculated all statistical measures in each step (Supplementary file 2). For binary pathogenicity classification, we selected the threshold that maximised the Matthews correlation coefficient (MCC) for each tool. Accuracy ranged from 51% (FATHMM) to 84% (CADD) with a median value of 76%. The sensitivity ranged from 41% (FATHMM) to 100% (fitCons) with a median of 82.5%, while specificity ranged from 1% (fitCons) to 81% (BayesDel) with a median of 54%. High sensitivity and low specificity indicate that most predictors correctly predict the P/LP variants but misclassify the B/LB ones. MCC values ranged from 0.04 (fitCons), indicating almost random prediction, to 0.49 (CADD) with a median value of 0.32. CADD achieved the highest accuracy and MCC among all in silico tools tested, using the threshold maximising the MCC (>10.44 for pathogenic prediction), indicating good performance as a binary classifier for globin gene variants. However, this threshold is not optimal for predicting benign variants, with the achieved specificity (0.47) being below the median, hence misclassifying 101 out of 192 B/LB SNVs. Eigen-PC achieved the second highest MCC (0.44), sensitivity of 0.79, and specificity of 0.7, with decision threshold of 1.87.

**Table 1.** Results and performance comparison of in silico predictors with the optimal threshold based on MCC. #PV: number of predicted variants; Ac: accuracy; Se: sensitivity; Sp: specificity; MCC: Matthews correlation coefficient; LR+: positive likelihood ratio; LR-: negative likelihood ratio; 95% CI: 95% confidence interval.

Tool	Decision threshold	#PV	TP	FN	FP	TN	Ac	Se	Sp	MCC	LR+	LR +95% CI	LR-	LR- 95% CI
**BayesDel_addAF**	≥0.39	531	250	164	22	95	0.65	0.6	0.81	0.34	3.21	[2.19, 4.72]	0.49	[0.42, 0.57]
**CADD**	>10.44	886	655	39	101	91	**0.84**	0.94	0.47	**0.49**	1.79	[1.57, 2.05]	0.12	[0.08, 0.17]
**ClinPred**	>0.95	481	265	99	43	74	0.7	0.73	0.63	0.32	1.98	[1.55, 2.53]	0.43	[0.35, 0.53]
**Condel**	>0.3	481	331	33	76	41	0.77	0.91	0.35	0.31	1.4	[1.22, 1.61]	0.26	[0.17, 0.39]
**DANN**	>0.96	531	372	42	71	46	0.79	0.9	0.39	0.33	1.48	[1.28, 1.72]	0.26	[0.18, 0.37]
**Eigen-PC**	>1.87	531	329	85	35	82	0.77	0.79	0.7	0.44	2.66	[2, 3.52]	0.29	[0.23, 0.37]
**FATHMM**	≤–3.39	481	150	214	23	94	0.51	0.41	0.8	0.19	2.1	[1.42, 3.08]	0.73	[0.65, 0.83]
**fathmm-MKL**	>0.7	531	328	86	39	78	0.76	0.79	0.67	0.41	2.38	[1.83, 3.09]	0.31	[0.25, 0.39]
**GERP++**	>3.49	531	248	166	26	91	0.64	0.6	0.78	0.31	2.7	[1.9, 3.82]	0.52	[0.44, 0.6]
**integrated_fitCons**	>0.05	531	414	1	117	1	0.78	1	0.01	0.04	1.01	[0.99, 1.02]	0.28	[0.02, 4.51]
**LIST-S2**	≥0.75	344	246	28	39	31	0.81	0.9	0.44	0.36	1.61	[1.3, 1.99]	0.23	[0.15, 0.36]
**LRT**	<0.3	270	169	7	84	10	0.66	0.96	0.11	0.13	1.07	[1, 1.16]	0.37	[0.15, 0.95]
**MetaLR_score**	>0.8	481	251	113	42	75	0.68	0.69	0.64	0.29	1.92	[1.49, 2.47]	0.48	[0.39, 0.59]
**MetaSVM_score**	>0.6	481	260	104	39	78	0.7	0.71	0.67	0.34	2.14	[1.65, 2.79]	0.43	[0.35, 0.53]
**MutationAssessor**	>2.53	359	249	36	41	33	0.79	0.87	0.45	0.33	1.58	[1.28, 1.94]	0.28	[0.19, 0.42]
**MutationTaster**	>0.95	531	386	28	102	15	0.76	0.93	0.13	0.09	1.07	[0.99, 1.15]	0.53	[0.29, 0.95]
**MutPred**	>0.5	467	343	12	96	16	0.77	0.97	0.14	0.2	1.13	[1.04, 1.22]	0.24	[0.12, 0.49]
**phastCons17way**	>0.17	531	357	57	57	60	0.79	0.86	0.51	0.38	1.77	[1.46, 2.14]	0.27	[0.2, 0.36]
**phastCons30way**	>0.28	531	329	85	51	66	0.74	0.79	0.56	0.33	1.82	[1.48, 2.25]	0.36	[0.28, 0.47]
**phyloP100way**	>0.42	531	349	65	56	61	0.77	0.84	0.52	0.35	1.76	[1.45, 2.14]	0.3	[0.23, 0.4]
**phyloP30way**	>0.51	531	307	107	63	54	0.68	0.74	0.46	0.18	1.38	[1.15, 1.64]	0.56	[0.43, 0.72]
**PolyPhen-2**	>0.65	481	243	121	37	80	0.67	0.67	0.68	0.31	2.11	[1.6, 2.78]	0.49	[0.4, 0.59]
**PROVEAN**	≤–1.03	481	358	6	106	11	0.77	0.98	0.09	0.18	1.09	[1.02, 1.15]	0.18	[0.07, 0.46]
**REVEL**	>0.65	481	294	70	46	71	0.76	0.81	0.61	0.39	2.05	[1.63, 2.59]	0.32	[0.25, 0.41]
**SIFT**	<0.1	481	325	39	74	43	0.77	0.89	0.37	0.3	1.41	[1.22, 1.63]	0.29	[0.2, 0.43]
**SiPhy_29way**	>10.62	531	233	181	33	84	0.6	0.56	0.72	0.23	2	[1.48, 2.7]	0.61	[0.52, 0.71]
**VEST4**	>0.7	531	273	141	33	84	0.67	0.66	0.72	0.32	2.34	[1.74, 3.15]	0.47	[0.4, 0.57]
**Splicing prediction**														
**ada**	>0.5	56	47	3	1	5	0.93	0.94	0.83	0.68	5.64	[0.94, 33.8]	0.07	[0.02, 0.23]
**MaxEntScan**	Diff >2 and Per >5	54	50	2	1	2	0.95	0.96	0.67	0.55	2.88	[0.58, 14.31]	0.06	[0.01, 0.28]
**rf**	>0.6	56	47	3	1	5	0.93	0.94	0.83	0.68	5.64	[0.94, 33.8]	0.07	[0.02, 0.23]
**SpliceAI**	>0.65	663	35	23	1	604	0.96	0.6	1	0.75	365.09	[50.94, 2616.41]	0.4	[0.29, 0.55]

**Figure 4.**
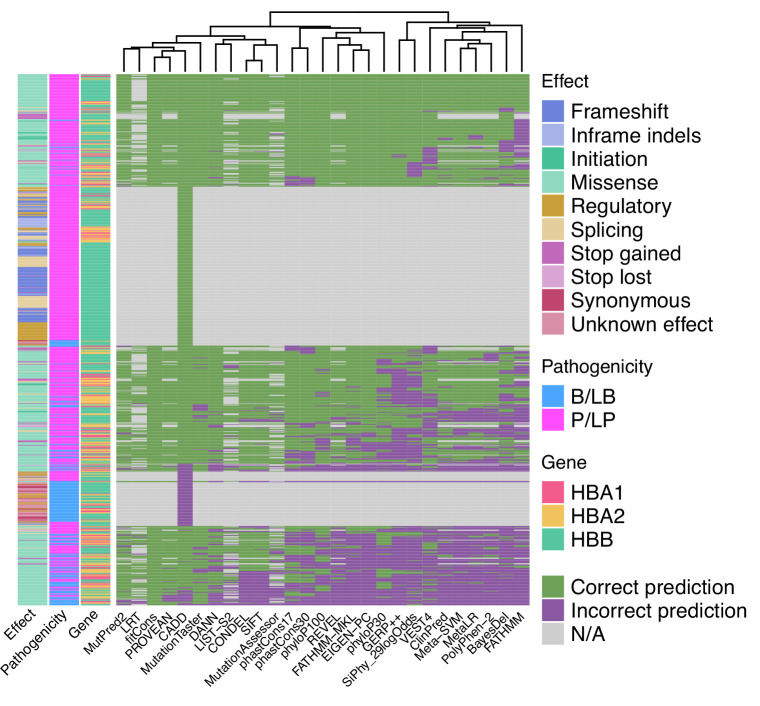
Heatmap illustrating the concordance and clustering of in silico tools with respect to the variant type and globin gene using the threshold that optimises the Matthews correlation coefficient (MCC), as shown in Table 1.

When used as binary predictors, the in silico tools were unable to reach the strength level required by the Bayesian framework (Tavtigian et al., 2018) to provide supporting evidence for variant classification. Although four tools (Eigen-PC, fathmm-MKL, VEST4, MetaSVM) achieved positive likelihood ratio (LR+) higher than 2.08 and negative likelihood ratio (LR-) lower than 0.48, required for supporting evidence strength for pathogenic and benign classification, respectively, their 95% confidence intervals (95% CI) extended beyond the above thresholds and, therefore, are not recommended alone for variant interpretation. Figure 4 shows a heatmap illustrating the extent of concordance among 27 in silico tools (excluding splicing tools) and clustering of the tools based on their concordance, using the thresholds that maximised the MCC (Table 1). Notably, we observe a high degree of concordance for P/LP variants in *HBB* (top of the heatmap), while there is a lower degree of concordance for variants in *HBA1* and *HBA2* (middle of the heatmap). The bottom part of the heatmap illustrates a higher discordance for B/LB variants in *HBA1* and *HBA2*.

### Performance of splicing predictors

Table 1 summarises the performance of in silico splicing tools using the threshold that maximised the MCC. With most SNVs affecting splicing regions of the globin genes annotated as P/LP, the performance of splicing tools cannot be compared reliably because of the limited number of negative examples in the dataset, that is, B/LB SVNs in splicing regions. Out of the four in silico tools tested, only SpliceAI provides a prediction score for variants that are not located near the canonical splicing sites. All splicing effect predictors displayed high accuracy, ranging from 93% (ada and rf) to 96% (SpliceAI), moderate to high sensitivity, ranging from 0.6 (SpliceAI) to 0.96 (MaxEntScan), and moderate to high specificity ranging from 0.67 (MaxEntScan) to 1 (SpliceAI). The MCC values ranged from 0.55 (MaxEntScan) to 0.75 (SpliceAI). SpliceAI achieved a high LR+ indicating strong performance in predicting SNVs disrupting splicing. The low number (≤5) of TN, FP, and FN in the predictions make the calculation of LRs for the remaining tools unreliable.

### Evaluation with different pathogenic and benign thresholds

**Figure 5.**
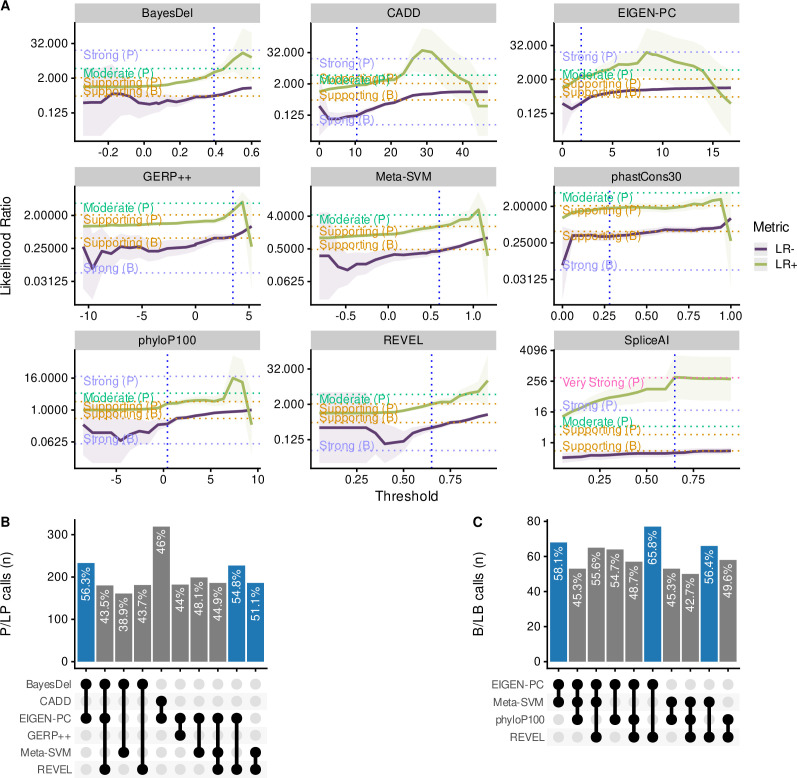
Comparison of the top performing in silico tools. (**A**) Likelihood ratios of the top performing in silico tools with variable threshold. Vertical dashed lines indicate the optimal threshold based on the highest Matthews correlation coefficient (MCC). (**B**) Concordant pathogenic/likely pathogenic (P/LP) calls by any given combination of in silico tools (among top performing tools) for pathogenic variants. (**C**) Concordant benign/likely benign (B/LB) calls by any given combination of in silico tools (among top performing tools) for benign variants. For Panels B and C, the concordance rate (i.e., variant assertion for all tools in the combination matches the expert annotation) is provided as text annotation on the bar chart. Only the first top 10 tool combinations based on concordance rate are shown, with the top three shown in blue.

We subsequently calibrated separate non-overlapping thresholds for pathogenic and benign prediction for each in silico tool to maximise both the percentage of variants correctly predicted by the selected threshold pairs that meet at least the supporting strength LR thresholds as defined by the Bayesian framework. More specifically, we filtered tools that achieved a lower bound 95% CI LR+ of 2.08 or higher for pathogenic prediction and an upper bound 95% CI LR- of 0.48 or lower for benign prediction. Figure 5A illustrates the changing LR values for the nine tools that reached these thresholds, while varying the decision thresholds. For these tools, we further finetuned the decision thresholds using smaller steps for the varying thresholds to maximise the number of correctly predicted SNVs. Furthermore, we tested the performance of all tools in different subsets of the dataset, including missense-only, non-missense, *HBB*, *HBA2,* and *HBA1* variants. Table 2 shows all threshold pairs that reach at least supporting level of evidence for both pathogenic and benign prediction in different SNV subsets. The full analysis for all thresholds and subsets is available in the Supplementary file 2 and the finetuning of the selected tools is available in Supplementary file 3.

**Figure 5—figure supplement 1.**
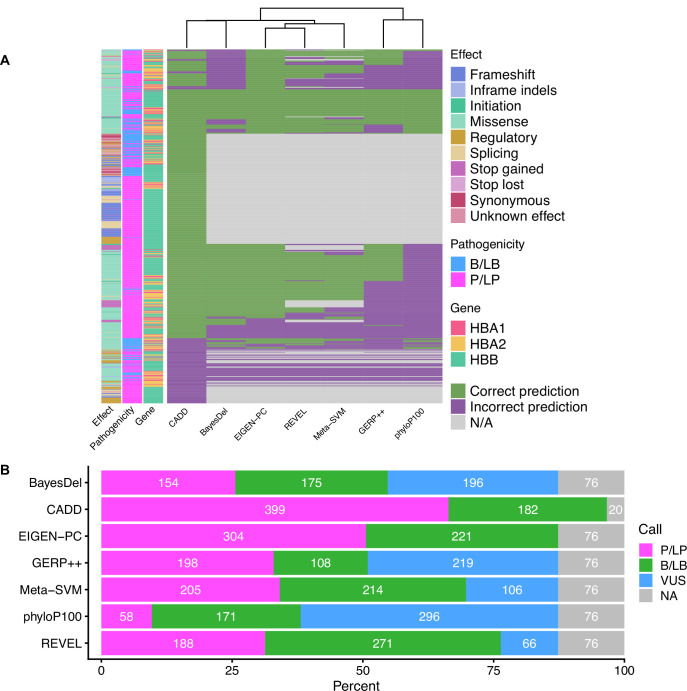
Concordance and VUS prediction of the top performing in silico tools. (**A**) Heatmap illustrating the concordance and clustering of the top performing in silico tools with respect to the variant type and globin gene, using the separate non-overlapping thresholds for pathogenic and benign prediction, as shown in Table 2. (**B**) Prediction of variants of uncertain significance (VUS) using thresholds for the full dataset (at supporting strength).

**Table 2.** In silico tools with pairs of non-overlapping thresholds that reach at least supporting evidence strength for both pathogenic and benign prediction. LR: likelihood ratio; CI: confidence interval; PV: predicted variants.

	Pathogenic prediction					Benign Prediction						
**Tool**	**Pathogenic threshold**	**Sensitivity**	**LR+**	**LR+ 95%** CI	**Strength (pathogenic**)	**Benign threshold**	**Specificity**	**LR-**	**LR- 95%** CI	**Strength (benign**)	**Correctly PV**	**% of correctly PV**
**All SNVs**												
**BayesDel_addAF**	≥0.39	0.6	3.21	[2.19, 4.72]	Supporting	<0.23	0.44	0.35	[0.26, 0.47]	Supporting	302	56.87
**CADD**	>25	0.39	8.27	[4.34, 15.75]	**Moderate**	≤21.75	0.78	0.42	[0.37, 0.48]	Supporting	418	47.18
**CADD**	>16.3	0.82	2.59	[2.1, 3.2]	Supporting	≤16.3	0.68	0.26	[0.21, 0.31]	Supporting	**703**	**79.35**
**Eigen-PC**	>1.9	0.79	3	[2.21, 4.07]	Supporting	≤1.9	0.74	0.28	[0.22, 0.35]	Supporting	415	78.15
**GERP++**	>4.22	0.44	4.33	[2.51, 7.49]	Supporting	≤0.15	0.35	0.32	[0.22, 0.46]	Supporting	225	42.37
**MetaSVM**	>0.81	0.55	3.25	[2.16, 4.89]	Supporting	≤0.46	0.6	0.38	[0.3, 0.48]	Supporting	272	56.55
**phyloP100way**	>7.32	0.15	17.8	[2.5, 127]	Supporting	≤0.8	0.57	0.36	[0.28, 0.46]	Supporting	130	24.48
**REVEL**	>0.77	0.63	3.05	[2.12, 4.4]	Supporting	≤0.7	0.69	0.38	[0.31, 0.47]	Supporting	309	64.24
**SpliceAI**	>0.3	0.67	58.12	[27.23, 124.03]	**Strong**	≤0.3	0.99	0.33	[0.23, 0.48]	Supporting	637	96.08
**Missense only**												
**BayesDel_addAF**	≥0.41	0.54	3.35	[2.2, 5.12]	Supporting	<0.22	0.44	0.32	[0.23, 0.45]	Supporting	241	51.72
**CADD**	>23.25	0.6	3.19	[2.17, 4.69]	Supporting	≤20.9	0.62	0.36	[0.28, 0.46]	Supporting	283	60.6
**Eigen-PC**	>1.9	0.78	2.93	[2.16, 3.98]	Supporting	≤1.9	0.74	0.3	[0.24, 0.38]	Supporting	**357**	**76.61**
**GERP++**	>4.22	0.44	4.27	[2.47, 7.4]	Supporting	≤–0.87	0.29	0.31	[0.2, 0.47]	Supporting	187	40.13
**MetaSVM**	>0.8	0.58	3.08	[2.09, 4.53]	Supporting	≤0.39	0.56	0.37	[0.29, 0.48]	Supporting	267	57.3
**phastCons30way**	>0.94	0.52	3.19	[2.09, 4.88]	Supporting	≤0.41	0.61	0.36	[0.28, 0.46]	Supporting	252	54.08
**phyloP100way**	>7.32	0.16	19.11	[2.68, 136.47]	Supporting	≤0.56	0.53	0.35	[0.27, 0.46]	Supporting	119	25.54
**REVEL**	>0.77	0.62	3.02	[2.09, 4.35]	Supporting	≤0.7	0.69	0.39	[0.32, 0.48]	Supporting	297	63.73
**Non-missense only**												
**CADD**	>11.5	0.93	8.62	[3.42, 21.77]	Supporting	≤11.5	0.89	0.08	[0.05, 0.11]	Supporting	350	92.84
**SNVs in *HBB***												
**BayesDel_addAF**	≥0.31	0.8	6.43	[2.23, 18.58]	Supporting	<0.31	0.88	0.22	[0.17, 0.3]	Supporting	210	81.08
**CADD**	>25.25	0.42	31.64	[4.5, 222.38]	**Moderate**	≤22.65	0.92	0.42	[0.37, 0.48]	Supporting	264	48.71
**CADD**	>10.8	0.94	3.26	[2.29, 4.64]	Supporting	≤10.8	0.71	0.08	[0.05, 0.12]	Supporting	494	91.14
**SNVs in *HBA1***												
**CADD**	>22.95	0.59	4.94	[2.29, 10.68]	Supporting	≤17	0.66	0.3	[0.19, 0.48]	Supporting	84	61.76

Notably, CADD is the only tool that reached a moderate level of evidence (LR+ lower bound 95% CI ≥4.33) for prediction of pathogenic variants (threshold >25), while BayesDel, Eigen-PC, GERP++, REVEL, MetaSVM, phyloP100way and CADD (with a lower threshold of 16.3) have also reached the supporting evidence strength. Importantly, CADD (at supporting strength), Eigen-PC and REVEL correctly predict the highest number of SNVs with 79.35%, 78.15%, and 64.24%, respectively. In addition, CADD and Eigen-PC achieve the highest sensitivity for pathogenic prediction with 0.82 (CADD threshold >16.3) and 0.79, respectively, as well as the highest specificity for benign prediction with 0.78 (CADD threshold ≤21.75) and 0.74, respectively. Moreover, SpliceAI reached strong level of evidence for splicing prediction (threshold >0.3), correctly predicting 96.08% of all variants, with a sensitivity of 0.67 and a specificity of 0.99.

When evaluating the performance of tools on the subset of missense variants, we identified eight tools (BayesDel, Eigen-PC, GERP++, MetaSVM, REVEL, CADD, phyloP100way, and phastCons30way) that reached supporting strength level. Eigen-PC, REVEL, and CADD achieved the highest percentages of correctly predicted SNVs with 76.61%, 63.73%, and 60.6%, respectively. Moreover, CADD performed well for non-missense variants where a single threshold of 11.5 produced an accuracy of 92.84%, while achieving supporting strength.

With regards to the gene-specific analysis, BayesDel and CADD performed well for the prediction of *HBB* variants using a single threshold and accuracies of 81.08% and 91.14%, respectively, with CADD achieving moderate strength for pathogenic prediction with a threshold of 25.25. Furthermore, CADD achieved supporting strength for SNVs in *HBA1*, whilst no tool reached the required LR thresholds for *HBA2*.

Figure 5B and C shows the concordance among the top performing tools of this study for pathogenic and benign prediction, respectively, using the recommended thresholds shown in Table 2 (full dataset; supporting strength thresholds). Although the overall concordance is low, some tools, such as Eigen-PC and REVEL, have higher concordance rates for both pathogenic (54.8%) and benign (65.8%) prediction. This is also demonstrated in the heatmap of Figure 5—figure supplement 1A illustrating the concordance of the top performing tools using the recommended thresholds. A higher degree of concordance is observed for P/LP variants in *HBB* (top and middle of the heatmap). The low concordance rate of the top performing tools is also reflected in the prediction of VUS (Figure 5—figure supplement 1B), where differences in the distribution of predicted pathogenicity classes are observed among in silico tools. Nonetheless, this will be further assessed when the pathogenicity status of these SNVs is clarified.

## Discussion

The main goal of this study was to assess the performance of in silico prediction tools in the context of haemoglobinopathy-specific SNVs and to provide evidence to the ClinGen Hemoglobinopathy VCEP for the most appropriate use of computational evidence in variant interpretation based on the ACMG/AMP guidelines. We evaluated the performance of 31 in silico predictors on a set of 1627 haemoglobinopathy-specific SNVs. The pathogenicity of these variants was assessed using a Delphi approach by haemoglobinopathy experts based on literature review and experimental evidence.

Our comparative analysis showed that, when used as binary predictors of pathogenicity, most tools have high sensitivity and accuracy but suffer from poor specificity. We show that binary classification results in low LRs for most tools and, thus, cannot be used alone based on the Bayesian framework for variant classification (Tavtigian et al., 2018). Instead, as we demonstrated in this study, stronger evidence is obtained when we trichotomised the problem by independently defining different non-overlapping thresholds for pathogenic and benign prediction of globin gene variants. This approach was previously described by other ClinGen VCEPs, evaluating sequence variants in other genes (Johnston et al., 2021; Pejaver et al., 2022) and, despite reducing the overall percentage of predicted variants, it increases the confidence of pathogenic and benign predictions because of higher LR values than the corresponding binary classifications. Our findings show that Eigen-PC, REVEL, and CADD performed well for predicting the functional effect of missense SNVs, while CADD was also a strong predictor of non-missense variants. Meta-predictors BayesDel and MetaSVM were also strong performers in our comparison, while GERP++, phyloP100way, and phastCons30way performed better among the conservation tools, albeit with a lower overall accuracy. Out of the four splicing prediction tools evaluated, SpliceAI performed better and produced the highest LR+ values reaching strong level of evidence. However, due to the low number of negative examples in our dataset for the other splicing tools evaluated, these results should be interpreted with caution. Our results show that SpliceAI is a reliable predictor of the splicing impact of SNVs in the globin genes.

In line with previous studies, our results reinforce the observation that several in silico predictors when utilised for binary variant classification perform differently for benign and pathogenic variants, by favoring the classification of variants as pathogenic (Ghosh et al., 2017; Gunning et al., 2021). The problem of false concordance has been widely reported in previous studies (Ghosh et al., 2017) and can be attributed to several reasons. Firstly, several in silico predictors do not directly predict the variant pathogenicity (i.e., the clinical effect) of a variant, but instead provide a prediction on how a variant affects a protein domain or reduces its catalytic activity, thus inferring it is damaging to protein function (Ernst et al., 2018; Ghosh et al., 2017; Shi et al., 2019; van der Velde et al., 2015). Moreover, low concordance may also arise due to variants with different allele frequencies, as studies have shown a strong correlation between specificity and allele frequency (Gunning et al., 2021; Niroula and Vihinen, 2019). In addition, data circularity can affect tools performance, with Ghosh R and colleagues showing that prediction efficacy is partly depended on the distribution of pathogenic and benign variants in a dataset (Ghosh et al., 2017).

In this study, we observed lower concordance for *HBA1*/*HBA2* compared to *HBB*. This can be attributed to the fact that the pathogenicity of variants in *HBA1*/*HBA2* is often less clear in the heterozygous state due to the number of genes involved (i.e., four copies of *HBA1*/*HBA2* compared to two copies of *HBB*). Therefore, a variant on *HBA1*/*HBA2* can be damaging at the gene level (e.g., reduced expression), with this effect not often being reflected on the phenotypic level in the heterozygous state. This is also reflected by the number of variants annotated with two stars in ClinVar, as previously highlighted by the ClinGen Hemoglobinopathy VCEP (Kountouris et al., 2021).

Notably, our analyses showed that meta-predictors, such as Eigen-PC, REVEL, and CADD, outperformed other tools. This category of algorithms uses the results of other individual prediction tools as features, thus integrating different types of information (e.g., conservation and sequence information) in the prediction model. The performance of meta-predictors is robust regardless of technical artifacts, levels of constraint on genes, variant type, and inheritance pattern mainly because their prediction scores are derived from weighing and combining multiple features and predictors (Ghosh et al., 2017; Gunning et al., 2021). However, as noted in previous studies, combinations of meta-predictors and any of the tools or conservation-based algorithms already incorporated in meta-predictors is not recommended, as it is more likely to yield discordant predictions and duplication in the analyses (Ghosh et al., 2017; Gunning et al., 2021).

The annotated pathogenicity of the variants in our dataset was based on criteria agreed by all co-authors of this paper. These criteria are not based on the ACMG/AMP framework, because there is currently no available standard for pathogenicity classification of globin gene variants. The ClinGen Hemoglobinopathy VCEP is currently piloting its ACMG/AMP specifications, which can be used for variant classification in the future, thus potentially leading to reassessment of in silico predictors for globin genes variants. Nevertheless, the current classification reflects the current knowledge about the pathogenicity of the variants in our dataset, agreed by experts involved in the molecular diagnosis of haemoglobinopathies in five countries (Cyprus, Greece, Malaysia, Netherlands, and Portugal). A potential limitation is that some benign variants have not been observed in trans with both a β-thalassaemia variant and the Hb S variant and, therefore, their pathogenicity is assigned based on the current knowledge in the field. However, our approach is justified, because small numbers of true benign SNVs reflect the reality in clinical diagnostics, where pathogenic SNVs associated with clinical phenotypes are more easily interpreted than benign ones.

This study provides evidence for the selection of the most suitable in silico tools for the interpretation of SNVs in the globin gene clusters using the ACMG/AMP guidelines. Specifically, we provide the optimal thresholds for different tools that can be used under the PP3/BP4 criteria, including missense and splicing variant interpretation, while optimal thresholds for conservation-based tools are also critical for the application of criterion BP7. To our knowledge, this is the largest study evaluating the disease-specific application of in silico predictors in variant classification under the ACMG/AMP framework and its associated Bayesian framework. Our approach can be further expanded for the optimal calibration of thresholds of in silico tools in other genes and diseases, hence facilitating variant interpretation using the ACMG/AMP framework.

## Materials and methods

### Dataset

Figure 1 shows a schematic representation of the main steps of our methodology. SNVs were retrieved from the IthaGenes database of the ITHANET portal (Kountouris et al., 2017; Kountouris et al., 2014). The dataset includes all SNVs (≤50 bp) curated in IthaGenes (access date: 05/02/2021) located in *HBA1*, *HBA2,* and *HBB*, excluding (a) disease-modifying variants, (b) complex variants with multiple DNA changes found in cis, and (c) variants whose genomic location is unclear, such as α-chain variants identified by protein studies without identifying the affected α-globin gene.

Additionally, we queried ClinVar (access date: 05/02/2021) (Landrum et al., 2018) for SNVs with a two-star review status and gnomAD (access date: 05/02/2021) (Karczewski et al., 2020) for benign/likely benign SNVs using PopMax Filtering Allele Frequency greater than 1% in *HBA1*, *HBA2,* and *HBB*. Any missing SNVs were added to both IthaGenes and the dataset of this study. The final dataset included 1627 distinct SNVs. Finally, the dataset was further processed using the batch service of Variant Validator (Freeman et al., 2018) to validate the HGVS names and correct any annotation errors.

### Annotated variant pathogenicity

To enable the evaluation of in silico predictions, we subsequently annotated the pathogenicity of each SNV and compared it to the results of in silico predictors. Specifically, we used existing curated information on IthaGenes and further collected available evidence in scientific literature for each SNV in the dataset. The pathogenicity for each SNV was annotated using the following criteria:

- Pathogenic/likely pathogenic (P/LP)

SNVs that result in abnormal haematology or abnormal Hb properties, or sometimes causing disease (i.e., dominant), when detected in heterozygotes,
OR
Causes disease when observed in trans with an established pathogenic variant or in the homozygous state

- Benign/likely benign (B/LB)

At least three (independent) occurrences of the variant in heterozygous state without any change in the haematological parameters and Hb properties
OR
Not causing disease when observed in trans with an established pathogenic variant

- Variant of uncertain significance

All variants that do not meet the above criteria for benign/pathogenic or have conflicting evidence

The SNV pathogenicity annotations produced in the above step (henceforth denoted as initial classification) were subsequently further assessed and reevaluated by the experts. We used a Delphi approach (Dalkey and Helmer, 1963) to allow independent evaluation of the curated evidence for each variant. The pathogenicity of each SNV was independently assessed by two different groups of haemoglobinopathy experts, using evidence curated by the IthaGenes database or collected as part of this study. Then, the independent expert annotations were merged into one final consensus classification. In cases of disagreement, a consensus pathogenicity status was decided, after discussion among all experts, or the SNV was marked as a VUS. SNVs that have been directly submitted to IthaGenes by experts not participating in this study and without a peer-reviewed publication describing the methodology and results, have been also annotated as VUS. Figure 1—figure supplement 1 illustrates the changes in pathogenicity annotation after the expert evaluation, demonstrating that most changes involved variants that were initially classified as VUS and were reclassified as P/LP or B/LB in the final annotation. The final consensus pathogenicity classifications produced for all SNV in this study have been added to the IthaGenes database and was used throughout this study. After descriptive analysis of the full dataset, 601 SNVs annotated as VUS were filtered out of the dataset.

For the evaluation of tools predicting the impact of variants on splicing, we further annotated variants with respect to their effect on gene/protein function and assembled the following datasets:

1. Variants affecting splicing: all P/LP variants annotated to affect splicing or being in the splicing region of the transcript, excluding variants that are annotated as both missense and splicing and, therefore the mechanism of pathogenicity is ambiguous.
2. Variants not affecting splicing: all remaining variants in the dataset (P/LP and B/LB), excluding those annotated as both missense and splicing.

For SpliceAI, we selected the highest of the four Delta Scores provided as output, while for MaxEntScan we used two different thresholds as follows: (a) the absolute difference between the reference and alternative allele (denoted as Diff), and (b) the absolute percentage of change between the reference and alternative allele (denoted as Per) (Tey and Ng, 2019).

### In silico prediction tools

Thirty-one in silico predictors were compared in this study, as follows: ada (Jian et al., 2014), BayesDel (Feng, 2017), CADD (Kircher et al., 2014), ClinPred (Alirezaie et al., 2018), CONDEL (González-Pérez and López-Bigas, 2011), DANN (Quang et al., 2015), EIGEN-PC (Ionita-Laza et al., 2016), FATHMM (Shihab et al., 2013), FATHMM-MKL (Shihab et al., 2015), fitCons (Gulko et al., 2015), GERP++ (Davydov et al., 2010), LIST-S2 (Malhis et al., 2020), LRT (Chun and Fay, 2009), MaxEntScan (Yeo and Burge, 2004), Meta-SVM (Kim et al., 2017), MetaLR (Dong et al., 2015), MutationAssessor (Reva et al., 2011), MutationTaster (Schwarz et al., 2014), MutPred2 (Pejaver et al., 2020), PolyPhen-2 (Adzhubei et al., 2010), PROVEAN (Choi et al., 2012), REVEL (Ioannidis et al., 2016), rf (Jian et al., 2014), SIFT (Ng and Henikoff, 2003), SpliceAI (Jaganathan et al., 2019), VEST4 (Carter et al., 2013), phastCons (phastCons17way and phastCons30way) (Ramani et al., 2019), phyloP (phyloP100way and phyloP30way) (Ramani et al., 2019), and SiPhy_29way (Garber et al., 2009). Four of the tools are focused on predicting the splicing impact of a variant (ada, MaxEntScan, rf, and SpliceAI), while six tools produce conservation scores (GERP++, phastCons17way, phastCons30way, phyloP100way, phyloP30way, and SiPhy_29way). We selected in silico tools recommended by ClinGen and available in the ClinGen VCI (Preston et al., 2022), as well as additional established tools used in previous studies. We employed the online version of the Ensembl VEP (McLaren et al., 2016) and its dbNSFP (Liu et al., 2020) plugin (version 4.2a) to obtain the prediction scores of the variants in our dataset.

### Predictive performance assessment

Commonly used scalar measures were employed to compare the prediction accuracy of in silico tools, including specificity, sensitivity, and accuracy. All of them can be derived from two or more of the following quantities: (a) true positives (TP), the number of correctly predicted P/LP variants; (b) true negatives (TN), the number of correctly predicted B/LB variants; (c) false positives (FP), the number of B/LB variants incorrectly predicted as P/LP; (d) false negatives (FN), the number of P/LP variants incorrectly predicted as B/LB. Specificity is defined as the fraction of correctly predicted B/LB variants, sensitivity is the fraction of correctly predicted P/LP variants, and accuracy is the ratio of correct predictions versus the total number of predictions (Hassan et al., 2019).

Moreover, we used the MCC (Matthews, 1975) to compare the performance of in silico predictors. MCC ranges from –1 (i.e., always falsely predicted) to 1 (i.e., perfectly predicted) with a value of 0 corresponding to random prediction. MCC is considered one of the most robust measures to evaluate binary classifiers (Chicco and Jurman, 2020). Hence, in our analysis, the optimal threshold for binary classification was the one that maximised the MCC for each in silico tool.

Following the guidelines of a Bayesian variant classification framework (Tavtigian et al., 2018), LRs for pathogenic (LR+) and benign (LR-) outcomes were calculated for each tool to evaluate the evidence strength of their pathogenicity prediction using the odds of pathogenicity (OddsP) in the Bayesian framework. According to the Bayesian framework, the strength of OddsP for each evidence level was set as follows: ‘Very Strong’ (350:1), ‘Strong’ (18.7:1), ‘Moderate’ (4.33:1), and ‘Supporting’ (2.08:1).

### Comparative analysis

The analysis was separated into three parts. First, we performed descriptive analysis of the dataset, including variants annotated as VUS, based on the variant type, the variant effect on gene/protein function, the haemoglobinopathy disease group, thalassemia phenotype, molecular mechanism, and annotated pathogenicity. Subsequently, we removed variants annotated as VUS and we compared the 31 in silico tools as binary predictors of variant pathogenicity by selecting the threshold that maximised the MCC for each tool. For predictors whose output scores ranged from 0 to 1, we used thresholds with intervals of 0.05, whereas for predictors with scores falling outside this range, we set custom ranges based on the observed minimum and maximum scores in our dataset. Finally, we identified separate non-overlapping thresholds for prediction of pathogenic and benign effect as recommended by the Bayesian framework for variant interpretation (Tavtigian et al., 2018), by selecting thresholds passing the recommended LR+ and LR- thresholds, while maximising the percentage of correctly predicted variants for each tool. For tools passing the LR thresholds, we further finetuned the decision thresholds using smaller steps to optimise the prediction accuracy. Statistical analysis and visualisation of the results were performed using custom R scripts and the epiR package.

### Data availability statement

All data generated or analysed during this study are included in Supporting File 2 and Supporting File 3. Supporting File 2 provides the full dataset and subsets used as input in the analysis (sheet names starting with ‘Input’) as well as the results of the analysis (sheets starting with ‘On’). Supporting File 3 includes the finetuning analysis for specific tools and data subsets, as described in the manuscript.

We make the source code for evaluating the tools and generating the figures presented herein, freely available at https://github.com/cing-mgt/evaluation-of-in-silico-predictors, (Tamana et al., 2022 copy archived at swh:1:rev:c3d397be71733aaeaa3738c979899b1f23f7457f).

## Data Availability

All data generated or analysed during this study are included in Supporting File 2 and Supporting File 3. Supporting File 2 provides the full dataset and subsets used as input in the analysis (sheet names starting with "Input") as well as the results of the analysis (sheets starting with "On"). Supporting File 3 includes the finetuning analysis for specific tools and data subsets, as described in the manuscript. We make the source code for evaluating the tools and generating the figures presented herein, freely available at https://github.com/cing-mgt/evaluation-of-in-silico-predictors, (copy archived at swh:1:rev:c3d397be71733aaeaa3738c979899b1f23f7457f).

## Additional files

Supplementary file 1. The list of ClinGen Hemoglobinopathy variant curation expert panel (VCEP) members.

Supplementary file 2. Table with the dataset used in this study and the resulting scores obtained by the in silico predictors, divided into different sheets and subsets: all short nucleotide variants (SNVs), missense only, non-missense only, *HBB*, *HBA1*, and *HBA2*.

Supplementary file 3. Refined thresholds for the nine selected in silico predictors, divided into different subsets: all short nucleotide variants (SNVs), missense only, non-missense only, *HBB*, *HBA1*, and *HBA2*. Only decision thresholds passing the likelihood ratio (LR) criteria for supporting evidence are shown.

MDAR checklist
