# Supplementary material for: Evaluation of in silico predictors on short nucleotide variants in HBA1, HBA2, and HBB associated with haemoglobinopathies
Source: eLife. 2022 Dec 1;11:e79713. doi: 10.7554/eLife.79713 (PMC9731569; doi:10.7554/eLife.79713)
Supplement: Peer review history

# Editor report: Editor's evaluation

**DOI:** 10.7554/eLife.79713.sa0

**Authors:** Robert Baiocchi

**Affiliations:** https://ror.org/00rs6vg23 The Ohio State University United States

The more frequent use of next generation sequencing of individuals for diagnostic purposes has led to the identification of numerous single nucleotide variants (SNVs). Association of SNVs with likelihood of clinical significance is reliant on multiple criteria making in silico predictions generated by panels of experts increasingly relevant to the field. This study compares the performance of 31 computational tools in classifying pathogenicity of SNVs in the human adult globin genes, and proposes an improved approach to achieve balanced predictions. This paper is of interest to scientists and clinicians within the field of Haemoglobinopathies diseases.

# Decision letter

**DOI:** 10.7554/eLife.79713.sa1

**Affiliations:** https://ror.org/00rs6vg23 The Ohio State University United States; https://ror.org/00wqczk30 Pasteur Institute of Iran Islamic Republic of Iran

Our editorial process produces two outputs: (i) public reviews designed to be posted alongside the preprint for the benefit of readers; (ii) feedback on the manuscript for the authors, including requests for revisions, shown below. We also include an acceptance summary that explains what the editors found interesting or important about the work.

**Decision letter after peer review:**

Thank you for submitting your article "Evaluation of in silico predictors on short nucleotide variants in HBA1, HBA2 and HBB associated with haemoglobinopathies" for consideration by *eLife*. Your article has been reviewed by 2 peer reviewers, and the evaluation has been overseen by a Reviewing Editor and Mone Zaidi as the Senior Editor. The following individual involved in the review of your submission has agreed to reveal their identity: Mohammad Hamid (Reviewer #2).

The reviewers have discussed their reviews with one another, and the Reviewing Editor has drafted this to help you prepare a revised submission.

Essential revisions:

Reviewer #1 identified multiple issues that need to be addressed by the authors. Please provide a point-by-point statement in response to these comments. This may require substantial revision of the manuscript.

*Reviewer #1 (Recommendations for the authors):*

– Both the lists of annotations for the dataset of variants should be provided and the authors must also provide a comparison of the original database annotations and their revised annotations in the form of a figure panel or table. This will help determine whether the observed low specificity for in silico predictions was due to the revised annotations.

– All the classification benchmarks and parameters must be explored and presented in the results for the improved approach with separate pathogenic and benign thresholds in Table 2: The addition of accuracy, sensitivity, specificity and MCC will enable comparison with classification using the same pathogenic and benign thresholds in Table 1. This data is present in supplementary file 3 but the binary classification metrics for the tools and thresholds shown in table 2 should be displayed alongside.

– The authors must discuss why the performance of certain tools was better or worse than others to help other researchers not familiar with these studies obtain a better understanding of the tools. When the improved approach was applied, certain tools performed better than others for certain classes of variants. The reasons for this must be clearly explained and if not known, then an attempt must be made to determine them. This is in the interest of selecting appropriate tools for in silico prediction by other groups based on some knowledge of the underlying functioning of these classifiers.

– Concordance or discordance among the tools after setting separate thresholds (shown in Figure 3B and 3C) would be understood easier if presented as in supplementary figure 2. Why is there more concordance for HBB and less for HBA variants initially? And why is there low concordance after the improved approach? Is it also low for HBB or does it show the same pattern as before? Authors must discuss the likely reasons.

– If possible the authors must evaluate metapredictors separately since some of them like CADD take as input scores from other in silico tools also used in this comparison. Did metapredictors perform better in general and after the improvement?

– Please explain the rationale for the proposed improvement by setting separate decision thresholds for pathogenic and benign classification. Why focus on the likelihood ratio instead of MCC or the balance of accuracy, sensitivity and specificity? Why is increasing specificity at the expense of reduced sensitivity better in this case according to the authors' judgement?

*Reviewer #2 (Recommendations for the authors):*

Although this study was done in a small cohort of patients, I suggested that the paper be accepted as an original article.

# Reply: Author response

**DOI:** 10.7554/eLife.79713.sa2

Reviewer #1 (Recommendations for the authors):

– Both the lists of annotations for the dataset of variants should be provided and the authors must also provide a comparison of the original database annotations and their revised annotations in the form of a figure panel or table. This will help determine whether the observed low specificity for in silico predictions was due to the revised annotations.

We have now addressed this point. Please check our response in “Comment 2”.

– All the classification benchmarks and parameters must be explored and presented in the results for the improved approach with separate pathogenic and benign thresholds in Table 2: The addition of accuracy, sensitivity, specificity and MCC will enable comparison with classification using the same pathogenic and benign thresholds in Table 1. This data is present in supplementary file 3 but the binary classification metrics for the tools and thresholds shown in table 2 should be displayed alongside.

We have now included Sensitivity at the Pathogenic Threshold and Specificity at the Benign Threshold to Table 2. However, in the analysis where we trichotomise the problem, we do not deem MCC and specificity at the pathogenic threshold or sensitivity at the benign threshold to be informative and are rather misleading. All these metrics for the two independent binary predictors (pathogenic and benign) are available in Supplementary File 3. Please also check our response for “Comment 3”.

– The authors must discuss why the performance of certain tools was better or worse than others to help other researchers not familiar with these studies obtain a better understanding of the tools. When the improved approach was applied, certain tools performed better than others for certain classes of variants. The reasons for this must be clearly explained and if not known, then an attempt must be made to determine them. This is in the interest of selecting appropriate tools for in silico prediction by other groups based on some knowledge of the underlying functioning of these classifiers.

We have now addressed this point.

– Concordance or discordance among the tools after setting separate thresholds (shown in Figure 3B and 3C) would be understood easier if presented as in supplementary figure 2. Why is there more concordance for HBB and less for HBA variants initially? And why is there low concordance after the improved approach? Is it also low for HBB or does it show the same pattern as before? Authors must discuss the likely reasons.

We have now addressed this point.

– If possible the authors must evaluate metapredictors separately since some of them like CADD take as input scores from other in silico tools also used in this comparison. Did metapredictors perform better in general and after the improvement?

We follow the same analysis methodology for all tools included in the study, including meta predictors. We highlight that meta predictors are superior in predicting the pathogenicity of globin gene variants and we discuss possible reasons for this in a newly added paragraph in the discussion (pg. 19; lines 417-426).

– Please explain the rationale for the proposed improvement by setting separate decision thresholds for pathogenic and benign classification. Why focus on the likelihood ratio instead of MCC or the balance of accuracy, sensitivity and specificity? Why is increasing specificity at the expense of reduced sensitivity better in this case according to the authors' judgement?

One of the main objectives of this study is to provide evidence for the use of in silico predictors under the Bayesian ACMG/AMP framework. This framework provides specific LR threshold for each strength level in the framework. Achieving these thresholds can decrease the overall specificity/sensitivity, but it increases the confidence that pathogenic or benign calls are correct. Please also see our response to “Comment 4”

Reviewer #2 (Recommendations for the authors):

Although this study was done in a small cohort of patients, I suggested that the paper be accepted as an original article.

We would like to thank the reviewer for the positive evaluation.
